# Tobacco Cessation on Prescription as a primary health care intervention targeting a context with socioeconomically disadvantaged groups in Sweden: A qualitative study of perceived implementation barriers and facilitators among providers

**DOI:** 10.1371/journal.pone.0212641

**Published:** 2019-02-21

**Authors:** Anne Leppänen, Solvig Ekblad, Tanja Tomson

**Affiliations:** Department of Learning, Informatics, Management and Ethics, Karolinska Institutet, Stockholm, Sweden; University of California San Diego School of Medicine, UNITED STATES

## Abstract

**Background:**

A new intervention, Tobacco Cessation on Prescription (TCP), has been developed in the Swedish primary health care (PHC) setting to address inequalities in health caused by tobacco use. It consists of counseling for at least 10 minutes, an individualized prescription of tobacco cessation treatment and follow-up on at least one occasion. TCP is currently being tested in clinical practice for the first time but there is a lack of knowledge about how it is perceived by health care providers.

**Aim:**

To explore PHC provider’s perceived barriers and facilitators of implementing TCP as an intervention targeting a context with socioeconomically disadvantaged groups in Sweden.

**Methods:**

Directed content analysis of transcripts from eight semi-structured interviews and one focus group interview with PHC providers with personal experience of TCP as informants. Data collection and analysis was guided by The Consolidated Framework for Implementation Research.

**Results:**

Perceived facilitators of implementing TCP were increased self-efficacy among the informants and involvement in the treatment among patients, which led to more intensive counseling and advice being taken more seriously by patients. Lack of resources, routines, and collaboration to work with tobacco cessation and lack of knowledge, motivation and self-efficacy among colleagues were perceived as barriers. Motivation and self-efficacy to quit was perceived as low among some patients, which was explained by low social support to quit, negative attitude and low adherence to treatment and tobacco being used as a coping strategy for life stress. Access to treatment for patients was limited by cost of treatment, long waiting times and focus on face-to-face counseling.

**Conclusion:**

TCP was perceived positively by the informants but access to treatment for patients was partly limited by how tobacco cessation services were organized. Lack of structural support, resources and differing attitudes among PHC providers need to be addressed to facilitate its implementation.

## Introduction

Tobacco use is the most common preventable cause of death globally [1], responsible for 100 000 new cases of disease and 12 000 deaths per year in Sweden [2]. It is unevenly distributed in the population [3], contributing to inequalities in health and worse health for socioeconomically disadvantaged groups who have a higher prevalence of tobacco use and are more difficult to reach with cessation support compared to less disadvantaged groups [4]. According to the United Nations Sustainable Development Goals good health and well-being for all and reduced inequalities should be prioritized policy areas [5]. In Sweden, policy goals to eliminate the preventable inequalities in health within a generation [6] and to reduce the prevalence of daily smoking to less than 5% by the year 2025 [7] have been adopted, suggesting that tobacco cessation for socioeconomically disadvantaged groups is a prioritized area in public health policy. This is also reflected in the national treatment guidelines for tobacco cessation where qualified counseling to all high-risk smokers, including adult daily smokers with low socioeconomic status, is given the highest priority [8].

In the Swedish health care system, primary health care (PHC) has the main responsibility for disease prevention and health promotion [9]. Since persons in socioeconomically disadvantaged groups visit PHC more often than those with a higher socioeconomic status [10], PHC has been identified as a relevant setting to improve the reach of tobacco cessation support to these groups. Data from 2014 suggests that 70% of all daily smokers want to quit and that a third of these want support from the health care system in doing so [11]. However, only 2.5% of PHC patients are identified as daily smokers compared to 9% in the general population [3] and only 0.5% are offered qualified counseling [11]. This could be explained by high workload and lack of staff, time and resources to work with health promotion in PHC [12,13]. To reduce inequalities in health, the Swedish Commission for Equity in Health recommends that PHC’s mission to work with health promotion should be strengthened, including providing the time, competence and resources required to support those in greatest need to change their health-related behaviors [14].

Recently, the perceived feasibility and optimal design of Tobacco Cessation on Prescription (TCP) was explored in the Swedish PHC context [15]. The results showed that TCP could be a helpful tool for both patients and providers, potentially facilitating a more structured and effective approach to tobacco cessation for socioeconomically disadvantaged groups than current treatment practices in PHC [15]. TCP has been developed based on the findings from this study, on the previously mentioned national guidelines for tobacco cessation and on the Swedish version of Physical Activity on Prescription (PAP). PAP is a treatment that has been found effective in increasing physical activity and improving health and quality of life [16]. It has been used in the Swedish health care system to prevent disease and promote health in the general population since 2001 [17]. Similar PAP schemes have been introduced in more than ten other countries, suggesting positive outcomes on adherence rates of physical activity [18]. Like PAP, but adjusted to the tobacco cessation context, TCP consists of a counseling session, a prescription and follow-up. In more detail, TCP is defined as tobacco cessation counseling for a minimum of 10 minutes, followed by an individualized prescription of tobacco cessation treatment including options for further counseling, pharmacotherapy and other measures for cessation, and follow-up on at least one occasion. If TCP is effective in decreasing tobacco use among socioeconomically disadvantaged patients it may reduce inequalities in health caused by tobacco use. The effectiveness of TCP is currently being evaluated in a randomized controlled trial (RCT) [19] but qualitative research is also needed to explore implementation determinants from the perspective of those that have to implement it [20]. This is important since it may have implications for the acceptability and use of TCP [21], informing the further development and implementation of the method [20]. Thus, the aim of this study was to explore PHC provider’s perceived barriers and facilitators of implementing TCP as an intervention targeting a context with socioeconomically disadvantaged groups in Sweden.

## Methods

### Study design

An exploratory qualitative study design was used based on directed content analysis [22] of transcripts from semi-structured interviews, guided by the Consolidated Framework for Implementation Research (CFIR) [23].

### Study informants and setting

PHC providers with personal experience of TCP and willingness to participate were purposefully sampled and invited to participate as informants in the study. Purposeful sampling was applied to access those with the best knowledge and experience of the subject of interest [24]. The informants were identified through previous participation in the intervention group in the ongoing RCT that is evaluating the effectiveness of TCP in 18 PHC centers located in socioeconomically disadvantaged areas in Stockholm County [19]. Eligible informants were informed about the study during a routine follow-up visit to the PHC centers in the RCT. The informants were invited to participate by the researchers at least six months after their initial involvement in the RCT. Based on the authors’ estimation, six months’ experience of TCP was considered sufficient for the informants to reflect on the perceived barriers and facilitators of implementing TCP for the given target group and setting. The informants were offered a 100 SEK gift certificate as an incentive to participate in the study. In total, eight informants from six PHC centers participated in the study. Another three providers had relevant experience of TCP but they were not invited to participate since they were unavailable at the time of data collection due to sickness absence or change of workplace. All informants were female registered nurses, responsible for tobacco cessation treatment at their PHC centers in the RCT. Additional informant characteristics are presented in Table 1.

**Table 1:** Informant characteristics.

Characteristic	Informants n = 8
Age (years)	
Mean	45
Range	31–64
Work experience (years)	
Mean	16
Range	2.5–42
Experience of tobacco use, n (%)	
Never user	4 (50)
Former user	3 (37.5)
Current user	1 (12.5)
Specialist training, n (%)	
None	3 (37.5)
Asthma/COPD	3 (37.5)
Diabetes	2 (25)
Education in tobacco cessation treatment^a^, n (%)	
Part of nurse education	3 (37.5)
Motivational Interviewing	4 (50)
Basic education (2 days)	2 (25)
Specialist education (>2 days)	1 (12.5)
Other	4 (50)
Previous experience of working with tobacco cessation, n (%)	
Yes	3 (37.5)
No	5 (62.5)

^a^more than one education could be reported by each informant

### Theoretical framework

Data collection and analysis was guided by CFIR to inform further development of TCP and to identify barriers and facilitators to its implementation. CFIR is a theoretical framework developed in the US that is based on 19 implementation theories, mainly from the health care sector [23]. It was chosen as the theoretical framework in this study since it may be used in the pre-implementation phase to identify potential determinants to implementation [23]. It consists of 26 constructs and 13 sub-constructs divided into five domains; intervention characteristics, outer setting, inner setting, characteristics of individuals involved and process of implementation [23].

Due to the comprehensiveness of CFIR it is not feasible to collect data on all the constructs included in the framework. The constructs with the highest relevance for a particular study should thus be prioritized. The choice of CFIR constructs in this study is based on the results from the previous study that explored the perceived feasibility and optimal design of TCP [15]. The results from this study identified expected benefits and challenges with TCP but also stressed the importance of aspects like competence, resources, guidelines, follow-up, self-efficacy and motivation to use the method [15]. The CFIR constructs that informed the data collection are presented in the S1 and S2 Appendices.

### Data collection

Data was collected in September to October 2017 and April 2018 through eight semi-structured individual interviews in conversational form that were based on questions from an Interview Guide Tool specifically developed to cover the constructs of CFIR [25]. The informants were asked about their experiences of, and views on, TCP to identify perceived barriers and facilitators for a potential implementation of the method. The questions from the Interview Guide Tool were freely adapted to the TCP context and translated into Swedish by the authors. According to the recommendations in the Interview Guide Tool, the interview guide was also supplemented with broad open-ended questions that were specific to the study [25]. The interview guide was then pilot tested on one representative from the target group prior to the start of data collection. As a result of the pilot test and the first interview included in the data collection, one question about economic incentives was removed from the interview guide since it was misunderstood by the informants and financial aspects were reflected upon in response to other questions in the interview guide. Examples of interview questions included were: “How do you perceive TCP compared to other treatment options for tobacco cessation at the PHC center/in PHC?” and “How would you perceive it if TCP was to be implemented at your PHC center?” The interview guide is presented in the S1 and S2 Appendices. A copy of the TCP prescription form was also provided as a reference during each interview. The prescription form is presented in the S3 Appendix.

As suggested by the authors, all individual interviews were conducted face-to-face in rooms ensuring privacy at the informants’ workplaces. The individual interviews were conducted by the project coordinator of the RCT (AL) who has a background in public health science and health promotion and who was involved in the development of TCP.

The eight informants that participated in the individual interviews were also invited to participate in a focus group interview (FGI) to discuss the preliminary findings from the individual interviews. This was done to enhance credibility of the analysis and to further inform the researchers’ understanding of the data. Four of the informants agreed to participate in the FGI. Reported reasons for non-participation were related to high workload at the PHC centers. The FGI was conducted face-to-face in a private room at the authors’ workplace in May 2018. It was moderated by the principal investigator (TT) who has a background in nursing, prevention and public health science and who led the development of TCP. The FGI was observed by the project coordinator (AL). The interview guide for the FGI is presented in the S4 and S5 Appendices.

All interviews (on average 70 minutes long) were conducted in Swedish, audio-recorded and transcribed verbatim by the project coordinator (AL). In total, the transcripts were 277 A4 pages long and took approximately 73 hours to transcribe. The interviews were complemented with field notes that summarized the content of the interviews and described each interview situation and setting. The field notes were used to facilitate the transcription process and to reflect on methodological aspects but they were not included in the data analysis. In addition, data on age, gender, education, tobacco use, work experience and experience/education in tobacco cessation was collected through questionnaires at the start of the RCT, confirmed when conducting the individual interviews and complemented by the informants if needed. Written informed consent was obtained from all informants before the start of each interview. Ethical approval to conduct the study was obtained from the Regional Ethical Review Board in Stockholm [ref: 2015/207-31].

### Data analysis

The manifest and latent content of the transcripts was analyzed in a directed content analysis [22] guided by CFIR [23]. Since the study was conducted prior to any formal implementation of TCP into the health care system, the process of implementation domain in CFIR was not represented in the data. The constructs that were represented in the data are operationalized in Table 2.

**Table 2:** CFIR domains and operationalization of the constructs and sub-constructs represented in this study.

Domains	Operationalization of constructs
***Intervention characteristics***	*Evidence strength and quality*: Perceived evidence supporting the belief that TCP will have desired outcomes. *Relative advantage*: Perceived advantage of implementing TCP versus an alternative solution. *Adaptability*: The degree to which TCP can be adapted to meet local needs. *Complexity*: Perceived difficulty of TCP, reflected by duration, scope and number of steps required to implement TCP. *Design quality and packaging*: Perceived excellence in how TCP is bundled, presented and assembled. *Cost*: Costs of TCP and costs associated with implementing TCP.
***Outer setting***	*Patient needs and resources*: The extent to which patient needs for tobacco cessation treatment, including barriers and facilitators to meet those needs, are known and prioritized by the PHC center. *Cosmopolitanism*: The degree to which the PHC center is networked with other external organizations. *External policy and incentives*: External strategies affecting implementation of TCP, including policy, guidelines, economic incentives and benchmark reporting.
***Inner setting***	*Structural characteristics*: The structural characteristics of the PHC center. *Networks and communications*: The nature and quality of social networks, formal and informal communications within the PHC center. *Culture*: Norms, values and basic assumptions at the PHC center. *Implementation climate*: Tension for change: Perceived need for change regarding tobacco cessation treatment at the PHC center. Compatibility: The fit between TCP and existing workflows, systems and values at the PHC center. Relative priority: Shared perception of the importance of implementing TCP within the PHC center. Learning climate: A climate in which: a) leaders express their own fallibility and need for assistance and input; b) team members feel that they are valued and knowledgeable partners c) individuals feel psychologically safe to try new methods; and d) there is sufficient time for reflection. *Readiness for implementation*: Leadership engagement: Commitment of managers to implement TCP. Available resources: Resources available to implement TCP. Access to knowledge and information: Ease of access to information and knowledge about TCP and related interventions.
***Characteristics of individuals***	*Knowledge and beliefs about the intervention*: PHC provider’s attitudes toward TCP and related interventions. *Self-efficacy*^a^: Individual belief in PHC provider’s capabilities to use TCP. *Individual stage of change*: The phase the PHC provider is in, as he or she progresses toward skilled, enthusiastic and sustained use of TCP. *Individual identification with organization*: How PHC providers perceive the PHC center and their degree of commitment to the PHC center. *Other personal attributes*: Other personal traits of PHC providers such as personal motivation, values and learning style.

^a^an individual’s belief in their ability to perform the behaviors needed to achieve desired outcomes [26]

The transcripts from all interviews were brought together to constitute the unit of analysis. The transcripts were read several times by AL, SE and TT to get an overview of the data. SE is a researcher who has a background in psychology and PHC with experience in qualitative research methods and research on disadvantaged groups. AL and SE coded the data independently by identifying and labeling passages in the text describing barriers and facilitators to the implementation of TCP. Random checks of the material were then conducted to enhance the credibility of the coding. Additional differences in the coding were reviewed by AL and discussed with SE and TT until consensus was reached. The codes were then deductively assigned to the constructs of CFIR by AL. Lastly the analysis was reviewed by all authors and discussed until a final categorization was agreed upon. Member checking of the preliminary results from the individual interviews was conducted with four of the eight informants during the FGI to further strengthen the credibility of the analysis. The data from the FGI was then added to the analysis by repeating the procedure above. Three to four quotes from the informants are presented for each domain in CFIR in the results to support the analysis and to highlight some of the main findings.

## Results

Several barriers and facilitators to the implementation of TCP were identified. The results are presented below using the domains in CFIR as subheadings. In the subsequent paragraphs the constructs and sub-constructs in CFIR are emphasized in italics.

### Intervention characteristics

Regarding *evidence strength and quality*, most of the informants reported positive outcomes of TCP among their patients, either by them quitting or dramatically reducing their tobacco use. At the same time, relapse and inability to quit was also acknowledged. A reported *relative advantage* of TCP was increased self-efficacy in providing cessation treatment among the informants. Most of the informants reported that the counseling became easier, more clear and comprehensive with TCP and that patients were perceived to be more involved in the treatment, taking advice more seriously compared to standardized pamphlets or oral advice alone. Still, some informants found TCP to be insufficient and requested other interventions for the patients such as information campaigns. Regarding *adaptability*, TCP was considered flexible as it could be individually adapted to the patient and supplemented with additional questions and materials if needed. It could also be used in many different ways, for example as a complement to individual or group counseling, as a checklist, reminder, summary or written agreement between the provider and the patient:

”It feels more like […] binding […] because it isn’t just me who has decided what it says on the prescription form but it’s really them [the patients] themselves-or we have decided together so it becomes like a support that they have this when they’re about to quit smoking. You can just go back and look at what you agreed on because there’s a lot of information and you talk about a lot of things. You often sit there and talk for more than like an hour and then it’s good if all the important things you’ve concluded are written down as well.” [Informant 4]

TCP was not considered *complex* but easy to use and fast to fill out. Despite this, time and experience was required to learn the method. Furthermore, the *design quality and packaging* of TCP was considered clear and beneficial, both in terms of its components and in what was included on the prescription form but there were some discrepancies in which parts of the prescription form that were found valuable by the informants. For example, one informant did not find the front page helpful while other informants thought that some of the questions for self-reflection on the back were difficult for the patients to answer.

Using the prescription form and Motivational Interviewing as a basis for the counseling, connecting the prescription to the patient’s health status and asking patients about their tobacco use and previous experiences of tobacco cessation during the counseling was considered beneficial. Waiting room advertisement, guidelines for how to use TCP and including the prescription form in the electronic medical record (EMR) were seen as facilitators. Not having TCP in the EMR was considered a barrier since the information then had to be documented twice:

“The disadvantage is that it [TCP] is not included in TakeCare [the EMR]. I want that. And to avoid scanning and adding it into the medical record [manually] […] One more task, that was one too many”. [Informant 7]

Regarding *cost*, TCP itself was not perceived as resource demanding but the cost of educating PHC providers was considered a barrier for PHC centers that lacked staff with this expertise. Still, tobacco cessation was considered cost-effective from a societal perspective and this was used as an argument to finance the implementation of TCP and to allocate more resources to tobacco control:

“There is very, very much money to be saved [on tobacco cessation], so you should be able to have money for this important work. […] If you can lower the costs, reduce the tobacco use, you should be able to finance this [TCP].” [Informant 7]

### Outer setting

*Patient needs and resources* were discussed to a great extent. The informants perceived tobacco use as high in the target group and that there was a need and demand for support among patients to quit. Access to treatment was facilitated by regular contact with the target group, and awareness of and trust in available treatment options among patients together with offered support from providers. Access to treatment could be further facilitated by written information in different languages, lower cost of treatment and improved access to telephone, video and group counseling according to the patients’ needs. It was considered beneficial if the patients had a long-term perspective, positive attitude towards treatment and motivation, self-efficacy and social support to quit. Experienced consequences of tobacco use, a positive experience/outcome of the treatment and a good relationship between the provider and the patient was also considered beneficial.

In general, the informants viewed TCP as a patient centered approach, appropriate for the target group and a support appreciated by the patients. At the same time, they reported that some patients also found TCP repetitive and difficult to read and fill out. Furthermore, the prescription form could be perceived as demanding for patients with low motivation to quit or redundant for patients with high motivation to quit.

Other patient-related barriers identified were lack of motivation and self-efficacy to quit, lack of knowledge about the risks of tobacco use and benefits of quitting, as well as negative attitude and experiences of treatment. Lack of knowledge, demand and trust in evidence-based treatment options and low adherence to treatment were also mentioned as barriers, as was long-term tobacco use, lack of consequences from tobacco use and weight gain:

“Some [patients] don’t want to start with Champix [varenicline] […], some don’t believe in coming here to talk. […] There’s no use in coming here just to talk, they say. It won’t help.” [Informant 1]

Lack of cessation support from some providers and fear of disappointing or being judged by a provider if a quit attempt was unsuccessful were other patient barriers reported. Access to treatment was further limited by a focus on patient visits since patients were sometimes unable to attend face-to-face counseling at the PHC center due to work and travel. The cost of visits and pharmacotherapy and long waiting times for patients were also identified as barriers to access treatment:

”Some [patients] don’t have time and some have poor economy and don’t want to pay for visits. Otherwise, you’d like to meet those who are about to quit smoking a bit more often […], but I too am often like […] fully booked for some time ahead. So that’s also a barrier sometimes that there isn’t time to meet again as soon as you’d like”. [Informant 4]

Peer pressure and exposure to tobacco in the social environment were also perceived as barriers for the patients. Moreover, many patients were described as disadvantaged in various ways with different health and social problems such as mental illness, loneliness, unemployment, low income and educational level, which could make it more difficult for them to quit. Tobacco use was seen as a way for the patients to cope with their feelings and their life situation:

“I believe very much that the cigarette is a friend to many [patients], a psychological comfort and that it’s something that many are afraid of losing in some way. They think that they can’t do it […] and loneliness I’ve also noticed is a driving force for many to smoke. They talk with each other, smokers, and then suddenly you become more social, many think. I always tend to ask the question “What is positive about smoking?” […] and then these things come up that it’s social […] and what else do they usually say? “It’s calming”, many answer. Not everyone has a mental illness […] but on the inside there’s still worry and loneliness and anxiety and so forth. There is among most people but they have found a way then to cope with it [by using tobacco].” [Informant 6]

Regarding *cosmopolitanism*, the PHC centers were externally linked to some other organizations, for example through cooperation with colleagues at other PHC centers and in dental care. They also had information exchange with pharmacies, the pharmaceutical industry, municipalities and other stakeholders in tobacco control such as the Swedish National Tobacco Quitline. To the latter some patients were referred for continued cessation treatment. Mentioned barriers were miscommunication with pharmacies and underutilization of the Swedish National Tobacco Quitline.

*External policies and incentives* that promoted the implementation of TCP included societal and political support for tobacco cessation, such as the Tobacco Endgame goal to reduce the smoking prevalence in Sweden to less than 5% by the year 2025. Clinical guidelines and quality performance indicators for tobacco cessation were considered facilitators. Reimbursement for related activities such as physical visits for tobacco cessation counseling and asthma/chronic obstructive pulmonary disease (COPD) clinics were also recognized as beneficial. Barriers identified were inability among nurses to prescribe some pharmacotherapies for tobacco cessation and lack of reimbursement for telephone counseling and sufficiently long patient visits:

”I often call to follow up with many [patients] but when you think about the financial part of a PHC center, telephone counseling also takes time but it doesn’t give the PHC center any money […] You don’t receive any financial reimbursement”. [Informant 4]

### Inner setting

*Structural characteristics* that supported the implementation of TCP were continuity in the work and some degree of autonomy among PHC providers responsible for tobacco cessation. Lack of these and staff turnover were identified as barriers. Regarding *networks and communications*, clear responsibilities and routines for tobacco cessation and effective communication and collaboration between different professions at the PHC center were considered facilitators. This included having staff responsible for tobacco cessation at the PHC center that colleagues could refer patients to.

Support from colleagues, redistribution of competing work tasks to colleagues, an active dialog on tobacco cessation and forums for information sharing were also seen as facilitators. Unclear responsibilities, lack of routines, communication, collaboration, referral of patients from colleagues and being alone with the responsibility for tobacco cessation at the PHC center were identified as barriers:

“It’s not clear like how we should do with tobacco cessation. […] I have appointments to offer but if I don’t get patients from other colleagues, then it’s difficult. […] You should have like clear routines or yeah, staff responsible for it [tobacco cessation] and time allocated for it and of course collaboration with others to find patients”. [Informant 5]

It was described by the informants as part of the *culture* at the PHC centers that mainly nurses worked with tobacco cessation. The informants perceived tobacco cessation treatment as part of their professional role as nurses but referring patients, for example to the Swedish National Tobacco Quitline, was not considered part of their traditional professional role. This was seen as a barrier. The informants considered it important that all providers at the PHC centers worked with tobacco cessation. This was the case at some PHC centers but in others it appeared to be less common that other professions than nurses were involved in this work or asked patients about their tobacco use. The tradition in PHC to focus more on disease treatment than disease prevention was also mentioned as a cultural barrier.

Regarding the *implementation climate*, there appeared to be a *tension for change* as the informants expressed a need for more tools, routines, continuity, networks and collaborations to work with tobacco cessation. They also expressed a need for more comprehensive and intensive counseling, more experience and knowledge about tobacco cessation among providers and more time, resources and focus on tobacco prevention in PHC. TCP was considered *compatible* with ongoing changes at the PHC centers, including the increasing focus on health promotion and disease prevention in PHC. It was considered well-aligned with previous working methods such as PAP and Motivational Interviewing and not considered to increase workload. Lifestyle habits, tobacco cessation and asthma/COPD were described to have a high *relative priority* at the PHC centers but tobacco cessation appeared to have a lower priority among colleagues, partly since more acute health conditions and work tasks often had to be prioritized. Ongoing quality improvement work and research at some of the PHC centers promoted a positive *learning climate*.

Regarding *readiness for implementation*, *leadership engagement* was facilitated by managers supporting staff, taking responsibility for and prioritizing tobacco cessation. Lack of leadership engagement was mentioned as a barrier by some of the informants. This was partly explained by a financial focus among managers. A major barrier mentioned repeatedly by the informants was the lack of *available resources*, including lack of allocated staff, time, physical space and financial resources to work with tobacco cessation:

”When I work at the asthma/COPD clinic you have-you should have two hours per thousand listed patients according to it [the guidelines for an asthma/COPD clinic] and there smoking cessation is not really included so I actually don’t have any time at all allocated for smoking cessation but I do have […] those patients anyway but really you’d need more time allocated for it. I think that’s mostly what it’s about because you can never do anything good about anything if the time doesn’t exist. That’s just the way it is.” [Informant 4]

“I would really like to take […] some patients per month [for tobacco cessation counseling] and follow up with them but […] in reality it’s not possible. We don’t have the time.” [Informant 8]

Having these resources and *access to knowledge and information* about tobacco cessation and TCP was expected to facilitate the implementation of TCP. Beneficial competencies mentioned were those of nursing assistants, registered nurses, district nurses, specialists in asthma/COPD, diabetes and tobacco cessation. Training in tobacco cessation as part of the university education to become a nurse and further training, for example through web-based courses, tutoring, written materials and self-studies, was also considered beneficial, as was previous experience of Motivational Interviewing and counseling on lifestyle habits and tobacco cessation. Documentation and follow-up of tobacco use and cessation among patients in the EMR was also considered a facilitator. Lack of staff specialized in tobacco cessation and lack of experience, knowledge and education in tobacco cessation, particularly among colleagues, were considered barriers.

### Characteristics of individuals

*Knowledge and beliefs* about TCP were generally positive among the informants. It was seen as a support and a tool for providers. Of the components included in TCP, the informants generally expressed high trust in pharmacotherapy and counseling but distrust in Motivational Interviewing was also reported. Lack of experience with TCP among colleagues was viewed as a barrier. Informants with experience and prolonged use of TCP reported high *self-efficacy* in using TCP. The more experience they had, the more confident they felt about using TCP:

”The first and the second visit, it was a bit uncertain because you didn’t know so much but now the visit actually goes faster and I feel more confident and safe when I talk [about tobacco cessation] because I know what I’m talking about […]. Before you felt a bit of rumbling [tingling] in your stomach when you had a patient who wanted to talk about tobacco but after a few visits you felt like”Now I know this” […] much better than before I should say.” [Informant 3]

Many informants acknowledged that it can be difficult to ask patients about their tobacco use and to motivate those with low motivation to quit. Less experience with TCP was often associated with a lower self-efficacy to use TCP among informants and their colleagues. Thus, the *individual stage of change* varied between informants. Most of the informants were enthusiastic about TCP and used it as part of their daily practice. They intended to continue their use of TCP after the end of the RCT and wanted to disseminate it to colleagues within and outside the PHC center but some informants had been interrupted in their work with TCP due to external factors and were thus in earlier stages of change. High workload, stress and poor working environment had negative effects on their *individual identification with organization*:

”At my other PHC center a much longer time would be needed for the implementation of TCP to take off since now, the [lack of] physical space has the highest priority and there you can’t cope with any changes, you just survive. That’s what I think it looks like outside [in PHC] as well. Some PHC centers are so tight that you can’t really handle any changes at all. […] You’re almost working as an emergency department.” [Informant 7]

Regarding *other personal attributes*, all informants highlighted the importance of tobacco cessation and had a personal interest and motivation to work with it. Working with tobacco cessation, collaborating with colleagues, experiencing positive outcomes among patients and receiving appreciation from patients was perceived as rewarding for the informants. They valued a non-judgmental approach in the counseling and often had their own routine for tobacco cessation treatment. They also mentioned personal abstinence from tobacco use and experience of tobacco cessation as facilitators for the implementation of TCP. In contrast, personal tobacco use and a judgmental approach among providers were seen as barriers. Lack of motivation and interest among some colleagues to work with tobacco cessation was also considered a barrier:

”I don’t think that any knowledge would help them [colleagues] if they don’t have an interest for it [tobacco cessation] because they wouldn’t want to attend such trainings. Then it becomes meaningless for them and even if they were like told or offered by the manager “You should go to this training. We need someone at the PHC center who knows this”, they’ll go but either way, when they have been to the training, to Motivational Interviewing for example […], they won’t do the work that’s needed because they’re not interested.” [Informant 5]

## Discussion

### Results

To the best of the authors’ knowledge, this is the first study to explore PHC provider’s personal experiences of working with TCP in clinical practice. Similar to reported clinical experiences of PAP [27], TCP was perceived to increase self-efficacy among providers and involvement in the treatment among patients, leading to more comprehensive counseling and advice being taken more seriously by patients. This indicates that a prescription approach may be effective in changing not only physical activity levels [16] but also tobacco use among patients. Still, skepticism towards TCP was reported by some informants with less experience, knowledge and self-efficacy in providing tobacco cessation treatment. Educating PHC providers in how to prescribe physical activity has been found to increase knowledge, self-efficacy and provision of counseling, and reduce the perceived impact of barriers [28]. Educating providers will therefore be important when implementing TCP.

Previous studies on PAP have identified support from colleagues and managers [29,30], clear routines and allocated time to work with health promotion as determinants to implementation [29–31]. These were also mentioned by the informants as facilitators to the implementation of TCP. However, lone responsibility for tobacco cessation presented particular challenges at the PHC centers when workload and staff turnover was high. In accordance with studies on the implementation of other health promoting activities in PHC [12,29,30], differences in interest, knowledge, experience and self-efficacy to provide counseling were reported among colleagues, which limited their ability to cover for the provider responsible for tobacco cessation if this provider was absent. Moreover, the provider responsible was sometimes assigned other work tasks or had to cover for their colleagues, which led to prioritization of competing activities. High workload and competing priorities have previously been identified as barriers to implement PAP in Sweden [30,31] and health promoting activities in other PHC settings [12]. Lack of allocated time to work with tobacco cessation was considered the most important barrier to implementing TCP.

At the patient level, the informants identified several barriers to quit that have previously been found specific for socioeconomically disadvantaged groups. These included low motivation, self-efficacy and social support to quit, underestimation of the harmful effects of tobacco use, negative attitude, low adherence and access to treatment [32]. Similar to results from previous studies [32,33], tobacco use was also described as a coping strategy for life stress. Furthermore, the informants reported that access to treatment was partly limited by how tobacco cessation treatment was organized at the PHC centers. In addition to long waiting times and some providers not offering support, much of the treatment was focused on individual face-to-face counseling since this was what the PHC centers were reimbursed for. This was a barrier for patients that were unable to attend physical visits at the PHC centers, a finding that has been reported elsewhere [34]. Visits and pharmacotherapy were also associated with high costs for the patients, which could affect their willingness to seek and complete treatment [32,35].

### Methodological considerations

A potential limitation in this study was that the interviewers were involved in designing TCP and instructing the informants in the intervention procedure in the RCT. Thus, social desirability bias [36] may have affected the informants' accounts regarding the intervention. Social differences between the researchers and the informants such as age, socioeconomic status, cultural background and language may also have led to misunderstandings or misinterpretations in the communication, data collection and data analysis [37]. However, independent coding of the material by a researcher not involved in the design of TCP and member checking of the preliminary results with four of the informants during the FGI strengthened the credibility of the analysis [38,39]. Best practice guidelines for qualitative research were also applied in the reporting of this study to increase trustworthiness [39].

A methodological strength of this study was that a comprehensive framework was used to guide the data collection and analysis. Although CFIR has been widely cited since it was first published in 2009, a recent systematic review found that few studies have previously applied it to inform both data collection and analysis and that only two studies have applied it prior to the implementation of an intervention [40]. The many constructs included in CFIR allowed for many determinants to be captured that otherwise could have been missed. The application of predetermined constructs could also enhance the transferability of the results. A limitation of CFIR was that the interaction between the constructs was not taken into consideration in the framework.

Since TCP is a new intervention that is being tested for the first time in clinical practice in the RCT and few PHC providers have experience of the intervention, the number of informants eligible to participate in this study was limited. Even if there was some variation in the length and depth of the interviews depending on the informants experience with TCP, the participating PHC providers were those with the best knowledge of TCP. The specificity of the aim and sample of the study and the use of theory suggests that the sample size was sufficient in producing the information power needed [41]. The informants reported differences in attitudes between them and their colleagues, suggesting that their views may be different from those of other PHC providers. Still, the informants were recruited from multiple PHC centers in different sizes representing both private and public providers as well as rural and urban areas in different parts of Stockholm County. The results are also consistent with earlier research on tobacco cessation among socioeconomically disadvantaged groups [32,33,35] and barriers to implementing PAP [29–31] and other health promotion activities in PHC [12], suggesting that some of the results may be transferable to similar contexts. A limitation was that PHC providers were used as proxies to explore patient’s views on TCP. Future studies should thus explore how patients themselves perceive TCP.

### Implications for policy and practice

Reducing inequalities in health caused by tobacco use [14] could be a means to achieve the United Nations Sustainable Development Goals (specifically target 3.4 which aims to reduce non-communicable diseases) [5] and related policy goals in Sweden [6,7]. If TCP is viewed positively by both providers and patients and shown to be effective in reducing tobacco use among socioeconomically disadvantaged groups, it may be relevant to implement as a complement to standard practice for tobacco cessation treatment in Swedish PHC. Practical solutions such as integrating TCP in the EMR and training providers in how to use the prescription form will need to be addressed, but more importantly, knowledge, attitudes, resources and organizational support to work with tobacco cessation in PHC in general will need to be strengthened to facilitate such an implementation.

At the moment, tobacco cessation practices in the setting under study appear to be largely dependent on individual PHC providers. Previous research has found that health care professionals perceive the curriculum in university to have an impact on their behavior [12]. Increased emphasis on health promotion and tobacco cessation in the university and subsequent continuous education of health professionals could thus improve knowledge and attitudes toward tobacco cessation among PHC providers. Moreover, higher investment and introduction of formal requirements for tobacco cessation treatment in PHC could increase the relative priority and status of tobacco cessation treatment and perhaps promote a more systematic approach to this in PHC.

In addition, tobacco cessation interventions should be designed to maximize participation by vulnerable populations, taking barriers to access into account [33]. PHC services could be organized differently to better meet the needs of socioeconomically disadvantaged groups. For example, telephone counseling that has similar effectiveness as face-to-face counseling [42], could be offered to a greater extent. Free or subsidized treatment could also improve access to treatment for this target group [43]. Adapting interventions to increase access has been found valuable by patients and may have positive effects on cessation outcomes [44].

## Conclusions

TCP was perceived positively by the informants but access to treatment for socioeconomically disadvantaged groups was partly limited by how tobacco cessation services were currently organized. Lack of organizational support and resources, as well as differing attitudes and knowledge among PHC providers to work with tobacco cessation, will need to be addressed to facilitate its implementation.

## Supporting information

S1 Appendix Original interview guide for individual interviews in Swedish. (DOCX)

S2 Appendix Translated interview guide for individual interviews in English. (DOCX)

S3 Appendix Prescription form. (DOCX)

S4 Appendix Original interview guide for focus group interview in Swedish. (DOCX)

S5 Appendix Translated interview guide for focus group interview in English. (DOCX)
