# Supplementary material for: Tobacco Cessation on Prescription as a primary health care intervention targeting a context with socioeconomically disadvantaged groups in Sweden: A qualitative study of perceived implementation barriers and facilitators among providers
Source: PLoS One. 2019 Feb 21;14(2):e0212641. doi: 10.1371/journal.pone.0212641 (PMC6383914; doi:10.1371/journal.pone.0212641)
Supplement: S1 Appendix — (DOCX) [file pone.0212641.s001.docx]

# **S1 Appendix. Original interview guide for individual interviews in Swedish.**

| Introduktion |
| --- |

1. Hur arbetade ni med tobaksavvänjning på vårdcentralen innan ni började med Tobaksavvänjning på recept? Vilka behandlingsalternativ använde ni då?
2. Hur upplever du att det har varit att arbeta med Tobaksavvänjning på recept?
   1. Varför/hur kommer det sig? På vilket sätt?

| Interventionsegenskaper |
| --- |

**Relativ fördel**

1. Hur upplever du Tobaksavvänjning på recept jämfört med andra behandlingsalternativ för tobaksavvänjning på vårdcentralen/i primärvården?
   1. Vilka fördelar upplever du?
   2. Vilka nackdelar upplever du?

**Anpassningsförmåga**

1. Vilka förändringar i metoden bedömer du behövs för att Tobaksavvänjning på recept ska fungera på ett bra sätt på din vårdcentral?
2. Bedömer du att dessa förändringar är genomförbara? Varför eller varför inte?
3. Vilka delar borde behållas som de är?

| Yttre miljö |
| --- |

**Patientbehov och resurser**

1. Hur bedömer du att Tobaksavvänjning på recept möter behoven hos de patienter som besöker din vårdcentral?
   1. På vilket sätt kommer Tobaksavvänjning på recept att möta deras behov?
2. Vilka hinder för att delta i behandlingen bedömer du att patienter som besöker din vårdcentral kommer att möta?
3. Hur uppfattar du att patienter på din vårdcentral har upplevt att få Tobaksavvänjning på recept?
   1. Vad är deras syn på Tobaksavvänjning på recept?
   2. Beskriv/ge exempel.

| Inre miljö |
| --- |

**Kompatibilitet**

1. Hur upplever du att Tobaksavvänjning på recept stämmer överens med nuvarande arbetssätt och rutiner på vårdcentralen?
   1. Vilka problem eller komplikationer kan uppstå?

**Tillgängliga resurser**

1. Vilka resurser bedömer du behövs för att kunna införa Tobaksavvänjning på recept som en del av det rutinmässiga arbetet på din vårdcentral?
   1. Vilka resurser räknar du med? Vilka resurser har du fått? Vilka andra resurser skulle du vilja få?
   2. Vilka resurser kommer du sakna?

**Tillgång till kunskap och information**

1. Vilken typ av utbildning bedömer du behövs för att kunna använda Tobaksavvänjning på recept? För dig? För dina arbetskamrater (andra som arbetar på vårdcentralen)?
   1. Vilken typ av vidareutbildning behövs?

**Relativ prioritet**

1. Vilka andra projekt, initiativ och aktiviteter med hög prioritet pågår redan på din vårdcentral?
   1. Vilken prioritet bedömer du att införandet av Tobaksavvänjning på recept skulle ha jämfört med andra initiativ som pågår?
2. Vilka aktiviteter eller initiativ är viktigast för dig? För dina arbetskamrater (andra som arbetar på vårdcentralen)? För din arbetsgivare?
   1. Varför?

| Individuella egenskaper |
| --- |

**Kunskap och föreställningar om interventionen**

1. Hur skulle du uppleva det om man bestämde sig för att införa Tobaksavvänjning på recept på din vårdcentral?
   1. Varför?

**Tillit till den egna förmågan**

1. Hur trygg känner du dig att kunna använda Tobaksavvänjning på recept på din vårdcentral? Varför?
2. Hur är det då när det gäller dina arbetskamraters (andra som arbetar på vårdcentralen) förmåga att använda Tobaksavvänjning på recept? Varför?

| Avslut |
| --- |

1. Vad mer skulle du vilja tillägga/berätta som kan vara viktigt att veta om din upplevelse av Tobaksavvänjning på recept?
2. Hur har du upplevt den här intervjun?
