# Supplementary material for: Tobacco Cessation on Prescription as a primary health care intervention targeting a context with socioeconomically disadvantaged groups in Sweden: A qualitative study of perceived implementation barriers and facilitators among providers
Source: PLoS One. 2019 Feb 21;14(2):e0212641. doi: 10.1371/journal.pone.0212641 (PMC6383914; doi:10.1371/journal.pone.0212641)
Supplement: S2 Appendix — (DOCX) [file pone.0212641.s002.docx]

# **S2 Appendix. Translated interview guide for individual interviews in English.**

| Introduction |
| --- |

1. How did you work with tobacco cessation at the primary health care center before you started with Tobacco Cessation on Prescription? What treatment options did you use then?
2. How do you perceive it has been to work with Tobacco Cessation on Prescription?
   1. Why/how come? In what way?

| Intervention characteristics |
| --- |

**Relative advantage**

1. How do you perceive Tobacco Cessation on Prescription compared to other treatment options for tobacco cessation at the primary health care center/in primary care?
   1. What advantages do you perceive?
   2. What disadvantages do you perceive?

**Adaptability**

1. What changes to the method do you perceive are needed for Tobacco Cessation on Prescription to work well at your primary health care center?
2. Do you perceive that these changes are feasible? Why or why not?
3. What components should be kept as they are?

| Outer setting |
| --- |

**Patient needs and resources**

1. How do you perceive that Tobacco Cessation on Prescription meets the needs of patients that visit your primary health care center?
   1. In what way will Tobacco Cessation on Prescription meet their needs?
2. What barriers to participate in the treatment do you perceive that patients that visit your primary health care center will face?
3. How do you perceive that patients at your primary health care center have experienced Tobacco Cessation on Prescription?
   1. What is their view on Tobacco Cessation on Prescription?
   2. Please describe/give examples.

| Inner setting |
| --- |

**Compatibility**

1. How do you perceive that Tobacco Cessation on Prescription fits with current working methods and routines at the primary health care center?
   1. What issues or complications could arise?

**Available resources**

1. What resources do you perceive are needed to implement Tobacco Cessation on Prescription as part of the standard practice at your primary health care center?
   1. What resources are you counting on? What resources have you received? What other resources would you like to receive?
   2. What resources will you miss?

**Access to knowledge and information**

1. What kind of training do you perceive is needed to be able to use Tobacco Cessation on Prescription? For you? For colleagues (others that work at the primary health care center)?
   1. What kind of continued training is needed?

**Relative priority**

1. What other high-priority projects, initiatives and activities are already ongoing at your primary health care center?
   1. What priority do you perceive that the implementation of Tobacco Cessation on Prescription would be given as compared to other initiatives that are ongoing?
2. What activities are most important to you? For your colleagues (others that work at the primary health care center)? For your manager?
   1. Why?

| Characteristics of individuals |
| --- |

**Knowledge and beliefs about the intervention**

1. How would you perceive it if Tobacco Cessation on Prescription was to be implemented at your primary health care center?
   1. Why?

**Self-efficacy**

1. How confident do you feel about your ability to use Tobacco Cessation on Prescription at your primary health care center? Why?
2. How about your colleagues’ (others that work at your primary health care center) ability to use Tobacco Cessation on Prescription? Why?

| Ending |
| --- |

1. What else would you like to add/say that could be important to know about your experience of Tobacco Cessation on Prescription?
2. How did you perceive this interview?
