# Supplementary figures and images for: Tobacco Cessation on Prescription as a primary health care intervention targeting a context with socioeconomically disadvantaged groups in Sweden: A qualitative study of perceived implementation barriers and facilitators among providers
Source: PLoS One. 2019 Feb 21;14(2):e0212641. doi: 10.1371/journal.pone.0212641 (PMC6383914; doi:10.1371/journal.pone.0212641)

# **S3 Appendix. Prescription form.**

**
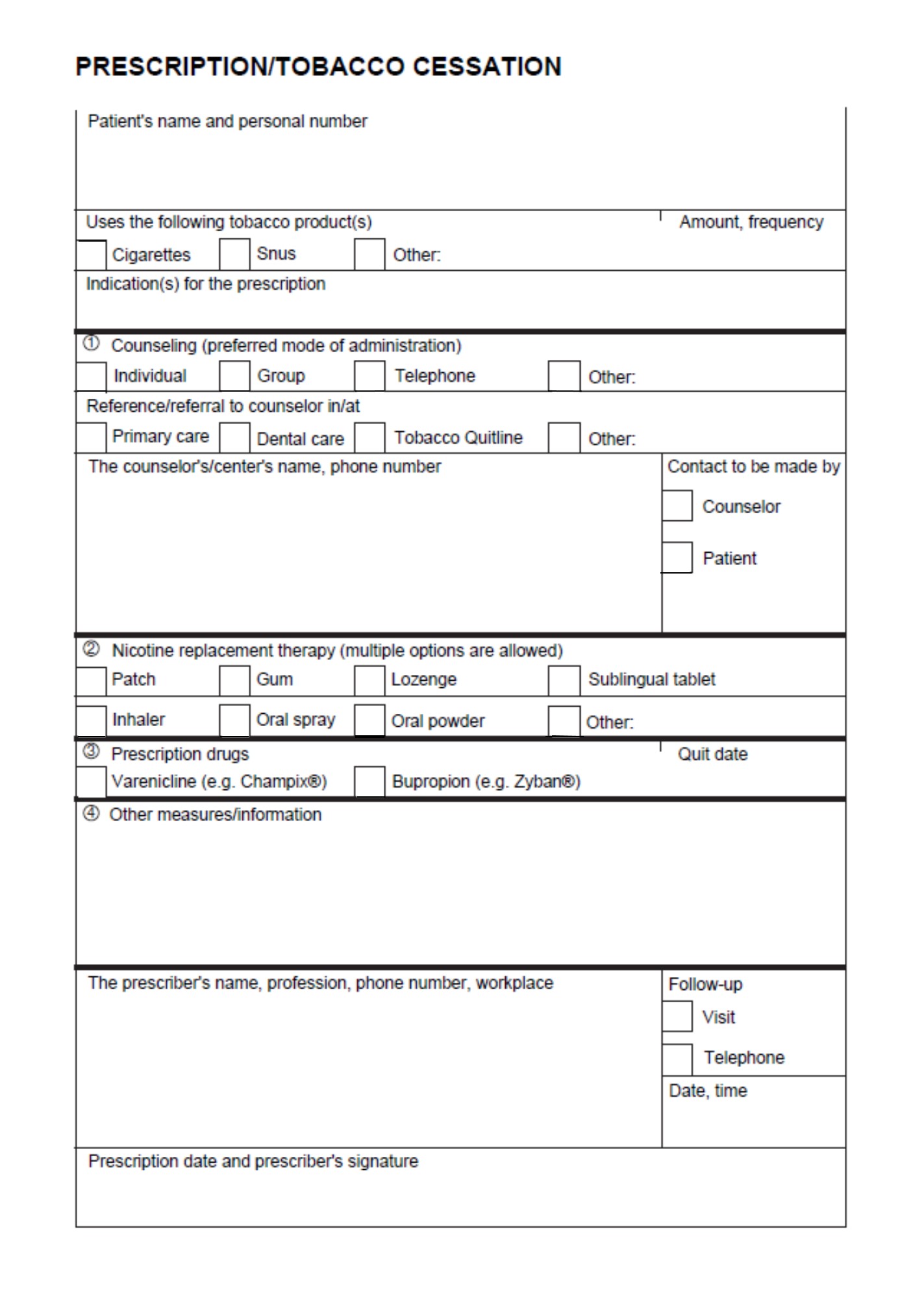
**

**
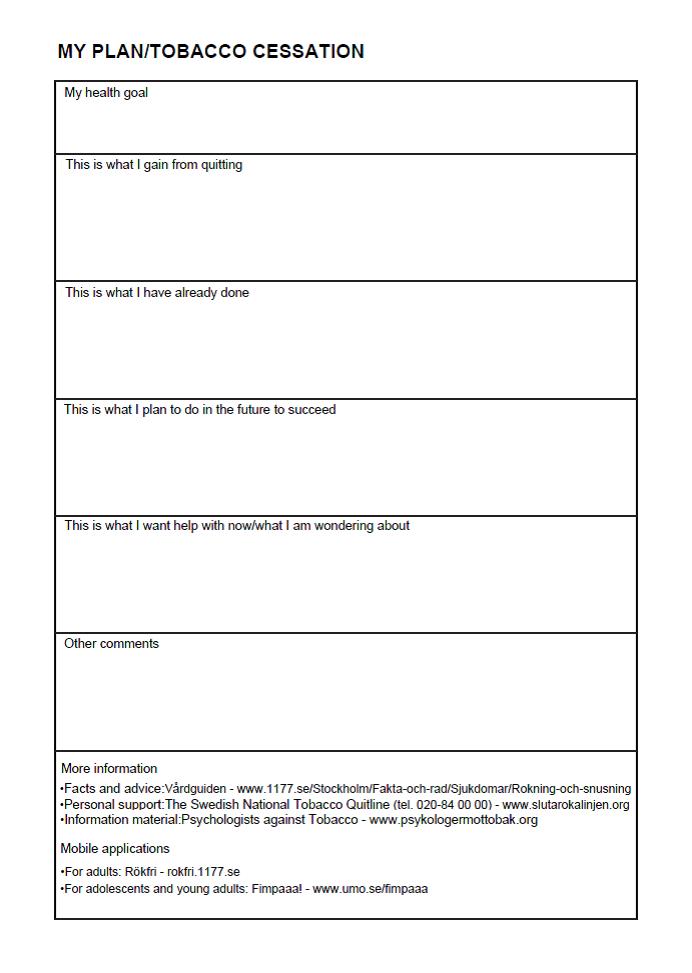
**

Supplement: S3 Appendix — (DOCX) [file pone.0212641.s003.docx]
