# Supplementary material for: Tobacco Cessation on Prescription as a primary health care intervention targeting a context with socioeconomically disadvantaged groups in Sweden: A qualitative study of perceived implementation barriers and facilitators among providers
Source: PLoS One. 2019 Feb 21;14(2):e0212641. doi: 10.1371/journal.pone.0212641 (PMC6383914; doi:10.1371/journal.pone.0212641)
Supplement: S4 Appendix — (DOCX) [file pone.0212641.s004.docx]

# **S4 Appendix. Original interview guide for focus group interview in Swedish.**

1. Vad tror ni att vi har kommit fram till efter intervjuerna med er?
2. Vad säger ni om det som vi har presenterat?
3. Vad känner ni igen från era egna erfarenheter?
4. Vad känns obekant utifrån era egna erfarenheter?
5. Vad är viktigast av det som tagits upp?
6. Vad saknar ni som kan vara viktigt att lägga till?
7. Hur upplevde ni den här gruppintervjun?
