# Supplementary material for: Tobacco Cessation on Prescription as a primary health care intervention targeting a context with socioeconomically disadvantaged groups in Sweden: A qualitative study of perceived implementation barriers and facilitators among providers
Source: PLoS One. 2019 Feb 21;14(2):e0212641. doi: 10.1371/journal.pone.0212641 (PMC6383914; doi:10.1371/journal.pone.0212641)
Supplement: S5 Appendix — (DOCX) [file pone.0212641.s005.docx]

# **S5 Appendix. Translated interview guide for focus group interview in English.**

1. What do you think we arrived at after the interviews with you?
2. What do you say about what we have presented?
3. What do you recognize based on your own experiences?
4. What feels unfamiliar based on your own experiences?
5. What is most important of what was brought up?
6. What do you miss that could be important to add?
7. How did you perceive this focus group interview?
